# Elastofibrome dorsal: étude rétrospective de 21 cas et revue de littérature

**DOI:** 10.11604/pamj.2013.16.52.2385

**Published:** 2013-10-14

**Authors:** Azzouz Haddan, Fouad Zouaidia, Siham Masmoudi, Abdelmonim Moumni, Mohammed Mouanis, Ahmed Jahid, Zakia Bernoussi, Najat Mahassini

**Affiliations:** 1 Laboratoire d'anatomie pathologique, hôpital Ibn Sina, Rabat, Maroc

**Keywords:** Elastofibrome, scapula, anatomie pathologie, chirurgie, elastofibroma, scapula, Anatomy pathology, surgery

## Abstract

L’élastofibrome est une tumeur bénigne du tissu mou survient essentiellement chez les personnes âgées de plus de 55 ans avec une prédominance féminine. Il survient électivement à l'angle caudal de la scapula (99%). Il est bilatéral dans 10% à 66% des cas. L'etiopathogenie de l'EF reste encore non élucidée. Il s'agissait d'une étude rétrospective au niveau du laboratoire d'anatomie pathologique d'Ibn sina de Rabat durant une période de cinq ans (2007- 2011), concernant 21 cas d'EF. Toutes ces lésions ont été diagnostiquées après coloration standard à l'HE et à partir des dossiers médicaux des patients, nous avons recueilli leurs données cliniques, radiologiques, leur prise en charge thérapeutique et leur suivi. Quizze des patients étaient de sexe féminin, 6 cas étaient de sexe masculin. Au moment du traitement l’âge moyen était de 57,6 ans. Chez 10 patients, la localisation de la tumeur était du côté droit, 6 cas du côté gauche et bilatérale chez 5 patients. La plupart des patients étaient asymptomatiques. La taille des tumeurs variait entre 5 et 14 cm de grand axe. En IRM, leur aspect était caractéristique et l’étude anatomo-pathologique avait confirmé le diagnostic chez tous les patients. L'elastofibrome est le diagnostic le plus probable quand il s'agit d'une localisation infra-scapulaire d'une masse du tissu mou. L'IRM est l'examen clé du diagnostic. Une éventuelle biopsie permettra d'exclure un processus tumoral malin et rassurer les patients asymptomatiques qu'aucun traitement chirurgical n'est nécessaire.

## Introduction

L’élastofibrome est une tumeur bénigne caractérisée histologiquement par l'association de fibres élastiques dystrophiques, de trousseaux denses de fibres de collagène, de tissu adipeux et de cellules fusiformes [1]. l'EF survient essentiellement chez les personnes âgées de plus de 55 ans avec une nette prédominance féminine [2–4]. Il atteint électivement la paroi thoracique dorsale à l'angle caudal de la scapula (99%). Il est bilatéral dans 10% à 66% des cas.

## Méthodes

Notre travail repose sur une étude rétrospective réalisée au laboratoire d'anatomie pathologique du centre hospitalier Ibn sina de Rabat, concernant 21 cas d'EF sur une durée de 5 ans étalée entre 18 Mai 2007 au 2 Aout 2011. Toutes les lésions ont été réséquées et adressées à notre laboratoire. A partir des dossiers médicaux, nous avons recueilli les données concernant les manifestations cliniques, radiologiques, les résultats histologiques, la prise en charge thérapeutique et le suivi à court et à moyen terme.

## Résultats

**Figure 1:**
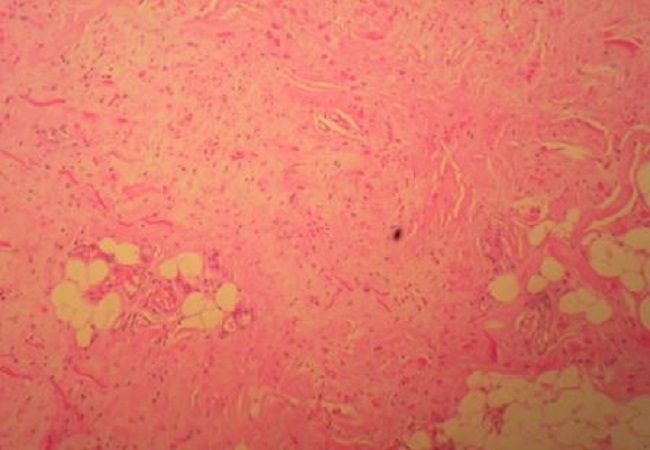
Microphotographie montrant des fibres élastiques morcelées ou en faisceaux au fort grossissement

**Figure 2:**
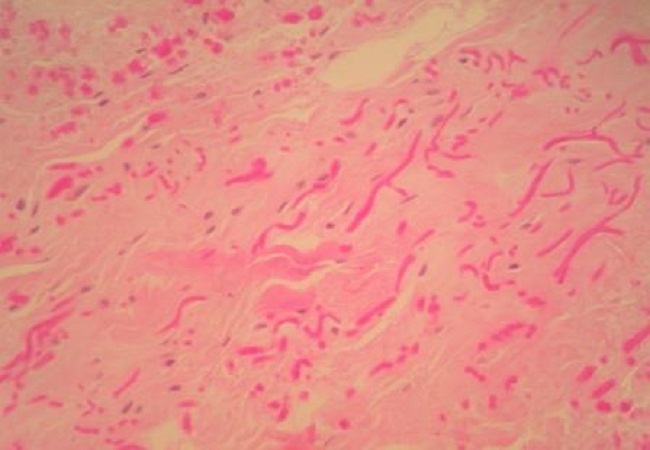
Microphotographie montrant le piégeage de tissu adipeux (blanc) au sein de la prolifération fibrocollagenique (rose) au faible grossissement

**Figure 3:**
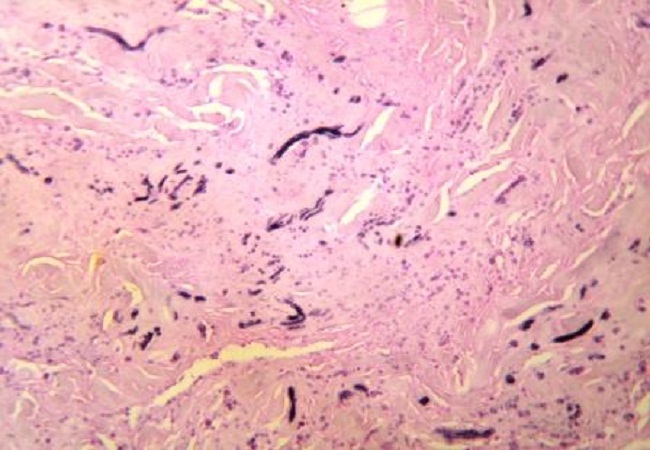
La coloration à l'orcéine fait ressortir les fibres élastiques, qui peuvent être de grande taille, ou prendre un aspect dit en "chapelet de perles"

Notre série regroupe 15 cas de sexe féminin et 6 cas de sexe masculin. Au moment du traitement l′âge moyenne était de 57.6 ans et variait entre 38 et 83 ans. Chez 10 patients, la localisation de la tumeur était du côté droit; dans 6 cas la tumeur était située du côté gauche; la localisation bilatérale a été constatée chez 5 patients. Dans la plupart des patients, les symptômes dominant étaient faits d'une simple gène fonctionnelle à la mobilisation de l’épaule. La taille des tumeurs variait entre 5 et 14 cm de grand axe. En imagerie, chez tous les patients ayant bénéficié d'une IRM, les marges des tumeurs étaient bien définies. Les tumeurs étaient situées en postéro-latéral de la paroi thoracique. Les masses formées par les EF étaient de contenu hétérogène. Le tissu fibreux présentait un signal faible en T1 et T2, similaire à celui du muscle. Des aires focales de tissu graisseux en signal intense en T1 et intermédiaire en T2. Les couches graisseuses se répartissaient en strates alternant avec les couches de type fibreux, déterminant des structures linéaires ou curvilignes, parallèles à la paroi thoracique. Absence de rehaussement de signale après injection de produit de contraste. L'examen échographique montrait un aspect fibrillaire et fasciculé de la lésion. Les patients consultaient tardivement vu que la majorité des cas étaient asymptomatiques. Aucun patient n'a signalé une limitation ou de gène à la mobilisation de l’épaule après traitement. Les donnés des patients sont rapportées dans le Tableau 1. Les données radiologiques étaient parfaitement corrélées à celles d'histopathologie: macroscopiquement il s'agissait le plus souvent de tumeurs mal limitées non encapsulées de consistance caoutcheuse. A la coupe leurs aspects étaient blanc jaunâtre dû au piégeage des lobules graisseux au sein de la trame fibreuse µ; aspect en damier »;. Sur le plan histologique, il s'agissait d'une tumeur non encapsulée imparfaitement limitée en périphérie, constituée d'une association de faisceaux collagéniques et de fibres élastiques irrégulières, disposées en faisceaux et fragmentées en petits globules éosinophiles (Figure 1). La trame fibrocollagénique est peu cellulaire et myxoïde. Du tissu adipeux mature est enchâssé dans cette prolifération (Figure 2). La coloration de l'orcéine met bien en évidence la présence de fibres élastiques caractéristiques, soit de très grande taille, soit présentant des aspects micronodulaires en chapelet de perles (Figure 3).

**Tableau 1:** tableau résumant les données médicales des patients

Cas	Age (ans)	Sexe	Comorbidité	Clinique	Côté/ bilatérale	Taille (cm)	Traitement	Evolution
1	83	Femme		Tumeur sous scapulaire bilatérale	Oui	8.5x6x2.5 5x5.5x2.5	chirurgie	Hématome postopératoire
2	70	Homme		Tuméfaction sous scapulaire droite douloureuse	Non Droit	11x8x2	chirurgie	favorable
3	65	Femme		Masse sous scapulaire gauche	Oui	9x8x2	chirurgie	favorable
4	51	Femme		Masse sous scapulaire droite	Non Droit	10x8x3	chirurgie	favorable
5	53	Femme		Tumeur sous scapulaire gauche	Non Gauche	8x7x5	chirurgie	favorable
6	65	Femme		Tumeur pariétale postérieure droite	Non Droit	11x9x3	chirurgie	favorable
7	55	Femme		Masse sous scapulaire droite	Non Droit	7x5x4.5	chirurgie	favorable
8	44	Homme	Diabète	Masse dorsale gauche	Non Gauche	10x9x2	chirurgie	favorable
9	69	Femme		Tumeur pariétale postérieure droite	Non Droit	8x6x3	chirurgie	favorable
10	61	Femme		Masse sous scapulaire droite	Non Droit	14x10x4	chirurgie	favorable
11	55	Femme		Masse sous scapulaire bilatérale	Oui	9x7x4 8x5x2	chirurgie	favorable
12	71	Femme		Masse sous scapulaire bilatérale	Oui	14x10x5	chirurgie	favorable
13	45	Homme		Masse sous scapulaire bilatérale	Oui	13x9x2.5	chirurgie	favorable
14	51	Femme		Masse postéro-latérale gauche + douleur mécanique	Non Gauche	8x7x5	chirurgie	favorable
15	58	Femme	Tuberculose pulmonaire	Tumeur pariétale sous scapulaire droite	Non droit	9x5x1.5	chirurgie	favorable
16	66	Femme		Masse pariétale sous scapulaire droite	Non droit	10x9x3	chirurgie	favorable
17	52	Homme	Adénocarcinome bronchique	Masse dorsale pariétale gauche	Non gauche	8x5x4	chirurgie	Perdu de vue
18	48	Homme		Masse pariétale sous scapulaire droite	Non droit	10x8x3	chirurgie	favorable
19	51	Femme		Masse pariétale gauche sensible	Non gauche	8x6x4	chirurgie	favorable
20	60	Homme	Adénocarcinome tubulo-papillaire bronchique	Masse pariétale	Non droit	8x5x2	chirurgie	Perdu de vue
21	38	Femme		Masse sous scapulaire droite	Non gauche	5x3,5x1	chirurgie	favorable

## Discussion

L’élastofibrome, bien que rare et d’évolution lente [3], doit être évoqué lorsqu'un patient rapporte une gêne fonctionnelle lors des mouvements de la scapula [4]. En effet, il n'est pas si exceptionnel, si bien qu'une série autopsique réalisée par Jarvi chez des patients de plus de 5ans le retrouve chez 24% des femmes et 11% des hommes [5]. Dans cette étude, les lésions mesuraient moins de trois cm, elles étaient donc trop petites pour être palpables ou pour avoir un retentissement clinique. Brandser et al. ont eux réalisé des scanners chez 258 sujets âgés asymptomatiques, et retrouvent des élastofibromes chez 2% des sujets [3]. Il est donc vraisemblable que la majorité des élastofibromes sont petits et cliniquement silencieux, à l'origine de la perception erronée d'une faible prévalence.

La pathogénie de l'EF dorsal reste encore flou, cependant les microtraumatismes à répétition, dû aux frictions entre omoplate et paroi thoracique, pourraient entrainer une hyperprolifération du tissu fibro-elastique réactive [6, 7]. Selon des séries, le rôle des microtraumatismes n'est pas encore établi vu que la plupart des auteurs n'ont pas fourni de données sur l'activité de leurs patients. On note une prédominance féminine avec un sexe ratio allant de 5/4 jusqu'au 13/1 [8], dans notre série le sexe ratio était de 15/6. Selon cette donne la notion de microtraumatisme ne peut être retenue comme facteur majeur de genèse d'EF [3, 4]. Des auteurs ont rapporté d'autres sites d'EF où il y a plus de friction au niveau de la valve tricuspide, des aisselles, des pieds, de la tubérosité ischiatique, de l'estomac et de médiastin; ce qui permet de maintenir la théorie avancée [9–15]. Plusieurs auteurs ont rapporté que l'insuffisance vasculaire pourrait être à l'origine de la dégénérescence élastosique [8]. Une prédisposition familiale peut se voir dans 30% des cas. Dans la grande série de Nagamine et al, les auteurs avancent l'origine héréditaire de la lésion [11]. Récemment, certains ont supposé que l’élastofibrome ne serait pas une pseudotumeur fibroblastique réactionnelle, mais un processus néoplasique monoclonal, avec une instabilité génomique. Ainsi, des modifications structurales, liées à une forte instabilité caryotypique, pourraient toucher presque tous les chromosomes [15]. Aujourd'hui, il est admis que l’élastofibrome est plus fréquemment rencontré chez les patients sollicitant leur ceinture scapulaire, même si le mécanisme n'est pas élucidé [10].

Les signes cliniques dépendent de la localisation et de la taille de la tumeur ainsi on aura soit une simple gène, une douleur mécanique ou un crissement lors de la mobilisation de l'omoplate. Dans notre série on avait deux cas qui présentaient une douleur mécanique. Dans 50% des cas les patients sont asymptomatiques ou présentaient un gène fonctionnel, ce qui explique la consultation à un âge tardive. L'EF survient principalement dans le côté droit et entre10% à 60% des cas il est bilatéral [8, 15]. Dans notre série la survenu de l'EF du côté droit était de 47%, de 29% du côté gauche et la localisation bilatéral était de 24%. La radiologie standard est non spécifique alors que l’échographie montre souvent un aspect fibrillaire et fasciculé de la lésion en rapport avec des stries hyperéchogènes parallèles à son grand axe. L’échographie peut représenter un outil rapide et moins couteux dans ce diagnostic [12, 13]. L'IRM est l'examen clé dans le diagnostic de ce type de lésion. Il montre un signal d'intensité faible comparable à celui du muscle. Les strates graisseuses montrent un signal intense responsable de l'hétérogénéité de l'image sans rehaussement après injection de produit de contraste. Les marges de la lésion sont bien définies [9, 14]. Chez tous nos patients les résultats radiologiques étaient compatibles avec ceux mentionnés ci-dessus. Le diagnostic différentiel radiologique inclut les sarcomes, les fibromatoses agressives, les lipomes et les fibromes, cependant ces lésions montrent un rehaussement après injection de produit de contraste sur l'IRM.

L′âge avancé des patients, la localisation typique, le sexe féminin font évoquer le diagnostic de l'EF. Devant ces cas et en plus d'image typique à l'IRM on peut s'abstenir de la biopsie [3]. D'autres auteurs recommandent un matériel tumoral pour confirmer ou établir un autre diagnostic. Le diagnostic histologique repose sur la présence de fibres d'allure élastosique souvent morcelés au sein d'une matrice collagénique. En microscopie électronique ce matériel éosinophile comporte fréquemment en son centre un tissu mature de fibre élastique et semble être secrété par des fibroblastes activées [8]. Ceux-ci confirme la thèse que le matériel élastosique provient des fibroblastes plutôt qu'une dégénérescence des fibres collagéniques. Des granulations denses cytoplasmique des fibroblastes semblent correspondre à de l’élastine ou aux précurseurs d’élastine [8]. L’évolution de nos patients était favorable, avec un recul de 2 ans en moyenne, compatible avec les données de la littérature.

## Conclusion

L'elastofibrome est le diagnostic le plus probable quand il s'agit d'une localisation infra-scapulaire d'une masse du tissu mou. L'IRM est l'examen clé du diagnostic. Chez les patients âgés ayant une localisation bilatérale et des résultats précis en matière d'imagerie on peut s'abstenir de la biopsie. Si non, la biopsie permettra d'exclure un éventuel processus tumoral malin et rassurer les patients asymptomatiques qu'aucun traitement chirurgical n'est nécessaire.
